# Supplementary material for: Synergic Renoprotective Effects of Combined ASC Therapy with RAAS Blockade in Experimental Advanced CKD
Source: Stem Cells Int. 2022 Mar 25;2022:5111782. doi: 10.1155/2022/5111782 (PMC8975629; doi:10.1155/2022/5111782)
Supplement: Supplementary Materials — Supplementary Figure 1: characterization of ASC at the 4th passage was performed by flow cytometry and cell plasticity tests: Cell population was positive for typical mSC biomarkers: CD44 (A), CD29 (B), CD90 (C), and CD105 (D) and negative for the pan leukocyte marker CD45 (E). 10,000 events were analyzed, of which 8,898 were viable (F). ASC phenotype before the plasticity tests is shown in (G). Chondrogenic differentiation was evidenced by the presence of sulfated matrix proteoglycans, highlighted in turquoise blue, and stained with Alcian blue (H); the osteogenic differentiation was verified by means of reddish calcium crystals stained with Alizarin Red (I), and finally, the adipogenic differentiation was verified by the presence of lipid droplets stained in red by Oil-red O (J). Microphotographs were taken under 400x magnification. Supplementary Figure 2: experimental protocol. On the 15th day after renal ablation, animals submitted to CKD induction were distributed into five experimental groups, randomized according to their basal UPE, as follows: basal CKD (N = 11), euthanized 15 days after 5/6 renal ablation; CKD (N = 13), kept untreated for 30 days; CKD + ASC (N = 15) that received a subcapsular injection of ASC at the 15th day of CKD and were followed until the 30th day after CKD induction; CKD + LOS (N = 12) that received 50 mg/kg/day of Losartan, diluted in drinking water, from the 15th to the 30th day after renal ablation; and CKD + ASC + LOS (N = 14) that received both the ASC subcapsular injection and the oral treatment with Losartan. Sham-operated animals (N = 11) were used as control. Supplementary Figure 3: in vitro experiments. (A) Illustrative microphotographs of immunofluorescence of cultured ASC, previously treated with as follows: normal rat serum (NRS), uremic rat serum (URS), or URS+10 μM of Losartan (URS + LOS). Cells positive for PCNA are stained in red. The total number of ASC/mm2 in each experimental treatment is represented in the bar gra [file 5111782.f1.docx]

**Synergic renoprotective effects of combined ASC therapy with RAAS blockade in experimental advanced CKD**

Marina PC Maires^1^, Krislley R Pereira^1^, Everidiene KVB Silva^1^, Victor HR Souza^1^, Flavio Teles^2^, Paulyana F Barbosa^2^, Margoth R Garnica^1^, Felipe M Ornellas^1^, Irene L Noronha^1^, Camilla Fanelli^1^*

^1^Renal Division, Department of Clinical Medicine, Faculty of Medicine, University of São Paulo, São Paulo, Brazil

^2^Renal Division, Department of Clinical Medicine, Faculty of Medicine, State University of Health Sciences, Alagoas, Brazil

**SUPPLEMENTARY MATERIAL**


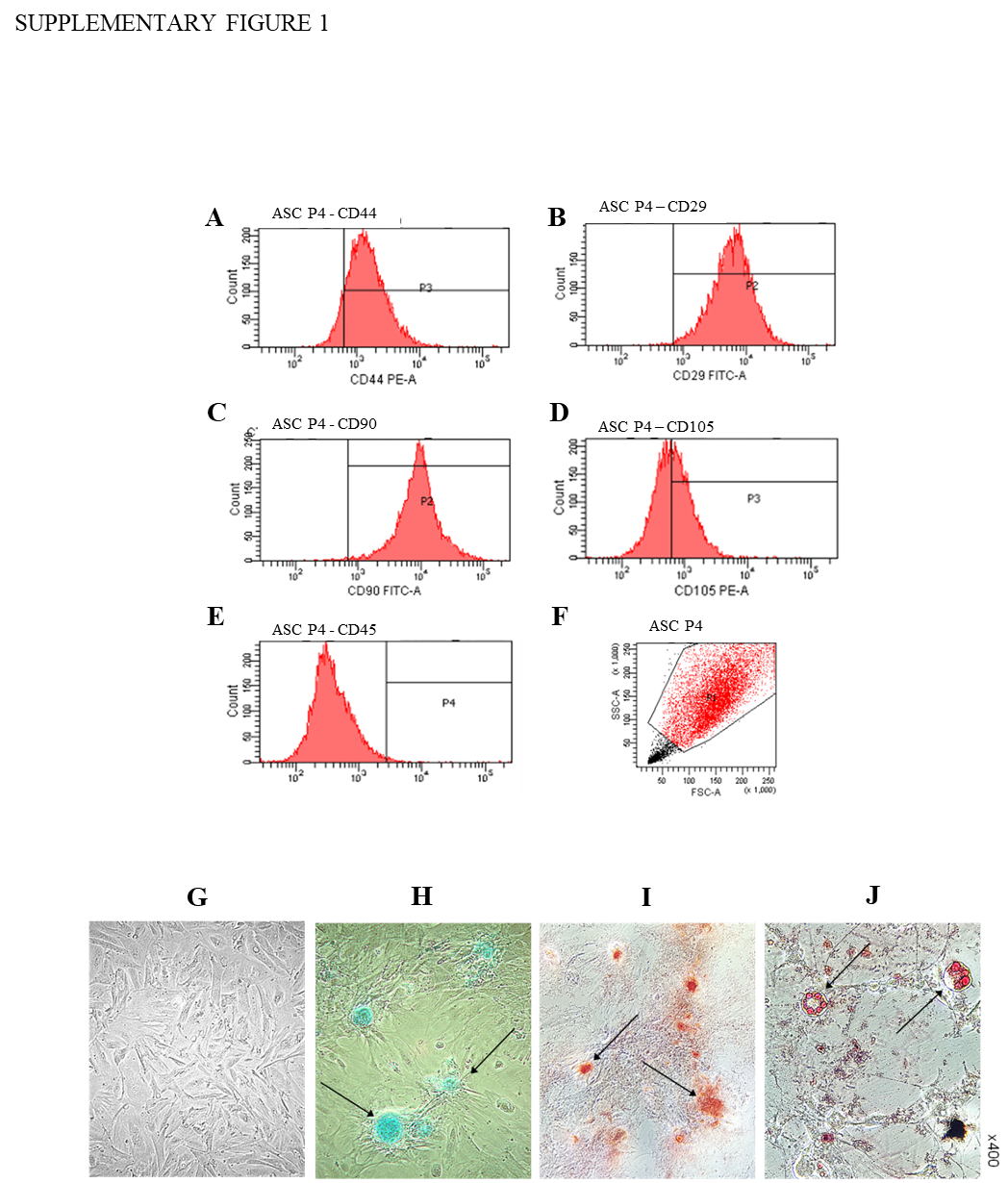
**SUPPLEMENTARY FIGURE 1**


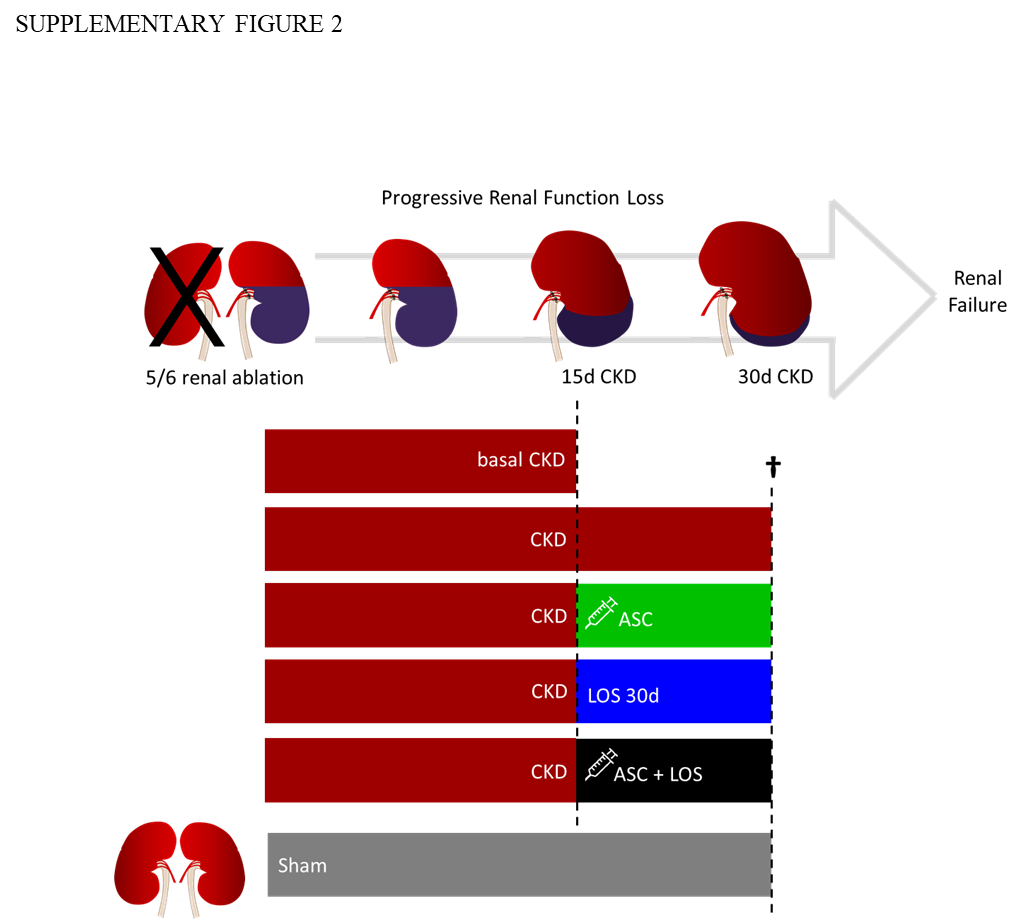
**SUPPLEMENTARY FIGURE 2**


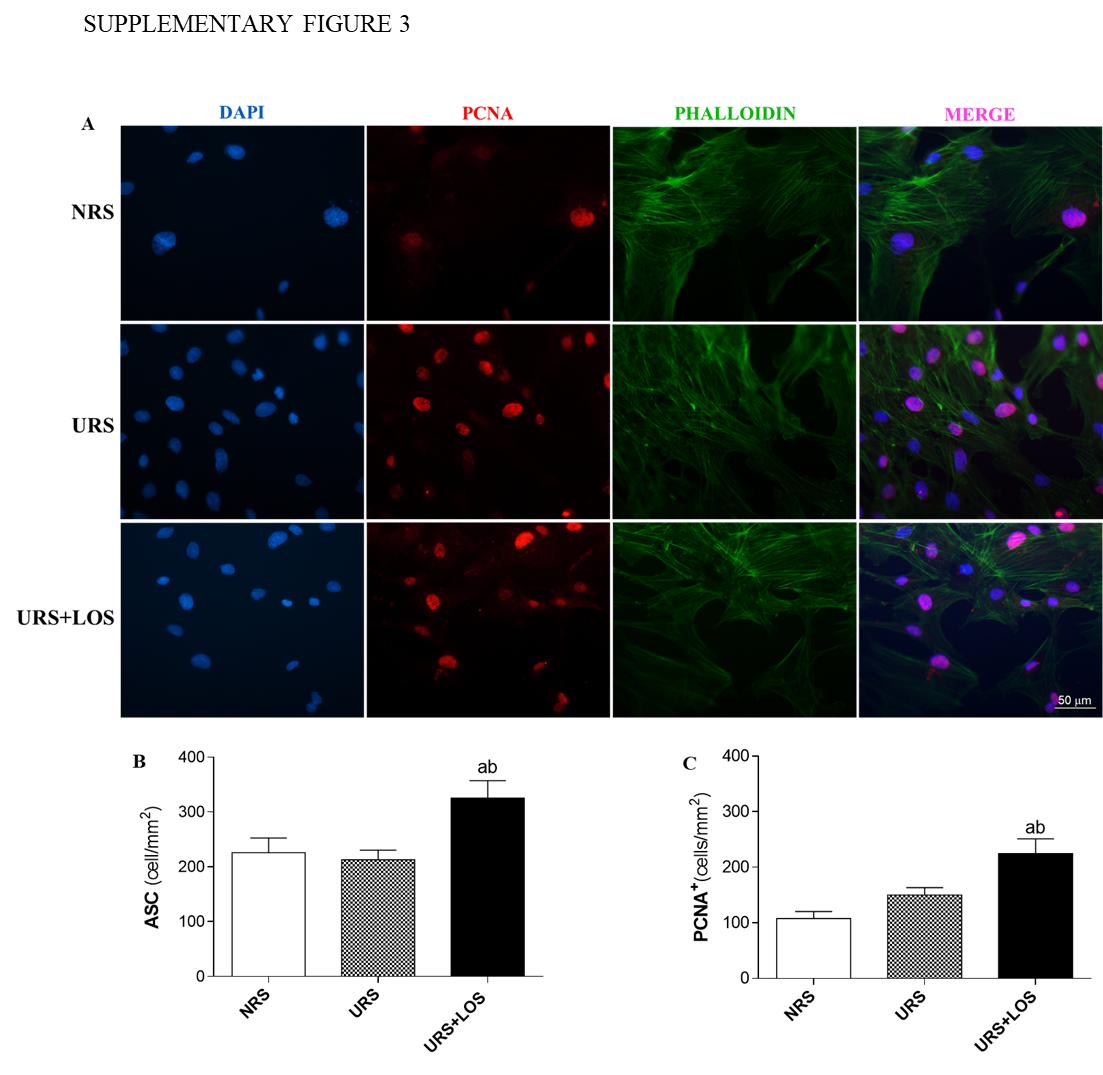
**SUPPLEMENTARY FIGURE 3**

**SUPPLEMENTARY FIGURE 4**

**
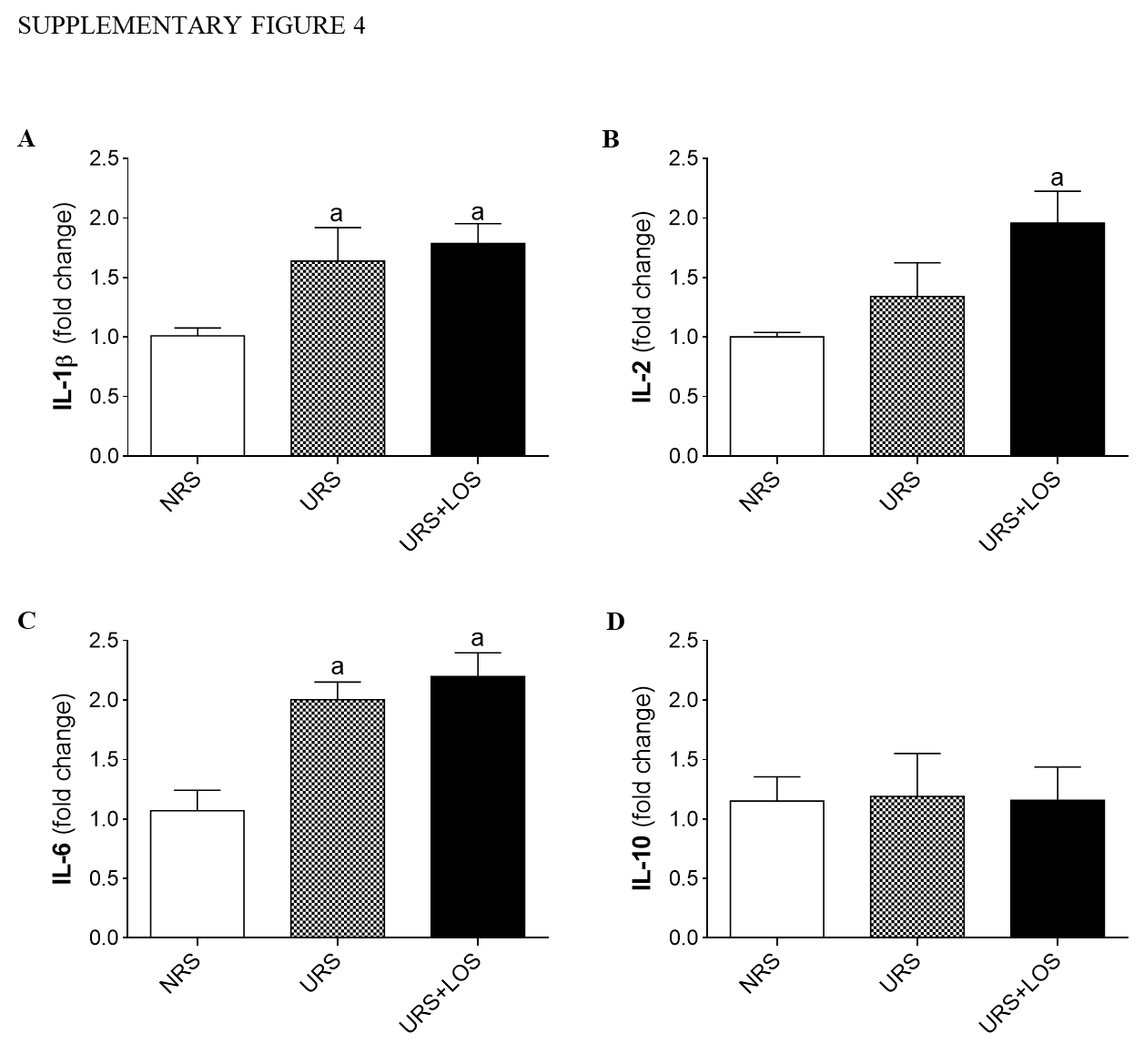
**

**SUPPLEMENTARY TABLE 1**

**
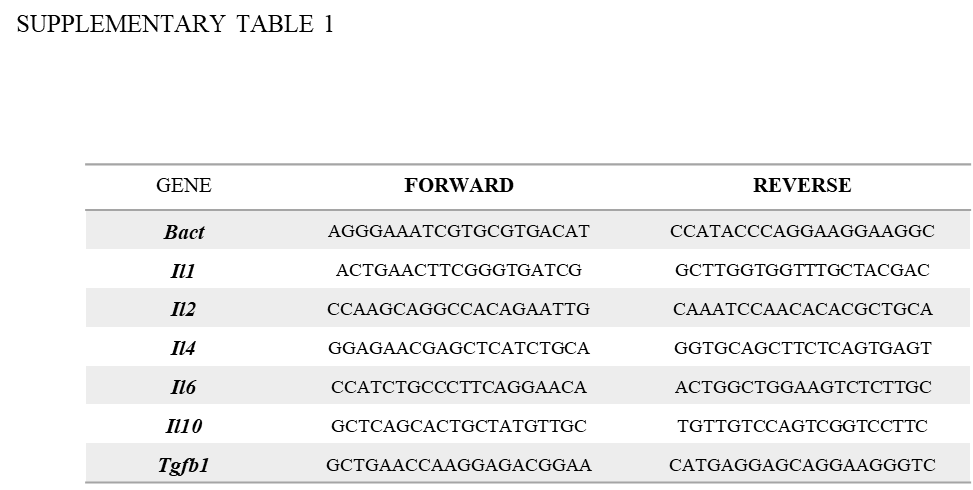
**


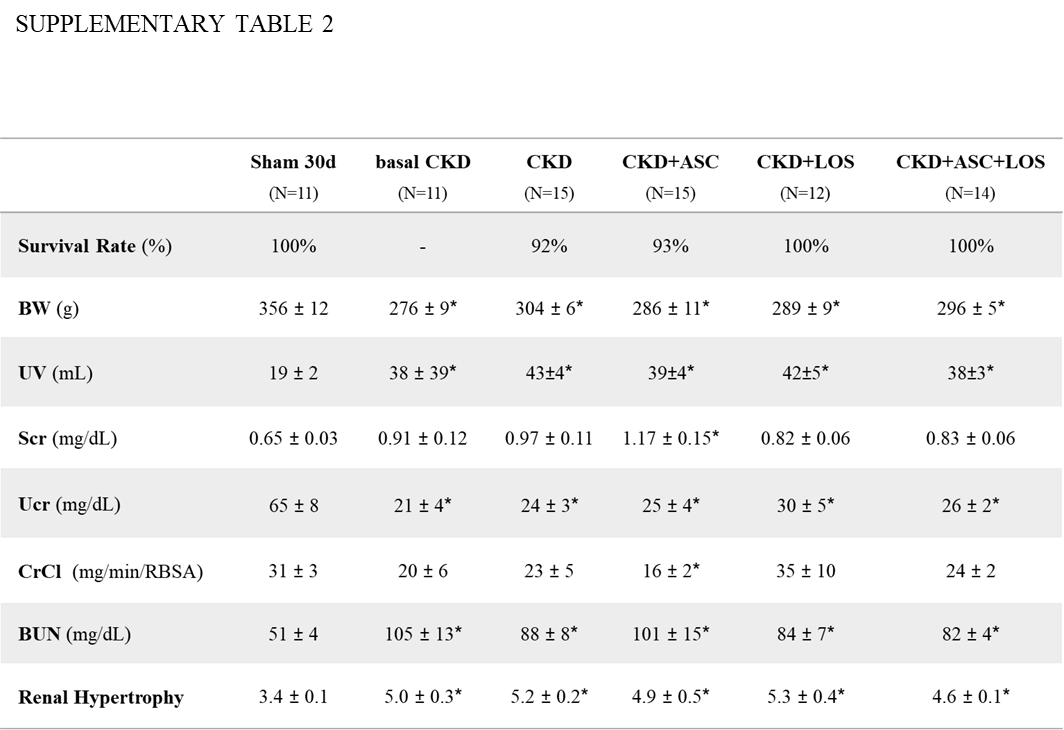
**SUPPLEMENTARY TABLE 2**

**SUPPLEMENTARY FIGURE AND TABLE LEGENDS**

**Supplementary Figure 1:** Characterization of ASC at the 4th passage was performed by flow cytometry and cell plasticity tests: Cell population was positive for typical mSC biomarkers: CD44 (**A**), CD29 (**B**), CD90 (**C**) and CD105 (**D**), and negative for the pan leukocyte marker CD45 (**E**). 10,000 events were analyzed, of which 8,898 were viable (**F**). ASC phenotype before the plasticity tests is shown in (**G**). Chondrogenic differentiation was evidenced by the presence of sulfated matrix proteoglycans, highlighted in turquoise blue and stained with Alcian blue (H); the osteogenic differentiation was verified by means of reddish calcium crystals stained with Alizarin Red (I) and finally, the adipogenic differentiation was verified by the presence of lipid droplets stained in red by Oil-red O (J). Microphotographs were taken under 400x magnification.

**Supplementary Figure 2:** Experimental Protocol. On the 15th day after renal ablation, animals submitted to CKD induction were distributed into five experimental groups, randomized according to their basal UPE, as follows: basal CKD (N=11), euthanized 15 days after 5/6 renal ablation, CKD (N=13), kept untreated for 30 days, CKD+ASC (N=15), that received a subcapsular injection of ASC at the 15th day of CKD and were followed until the 30th day after CKD induction, CKD+LOS (N=12), that received 50 mg/Kg/day of Losartan, diluted in drinking water, from the 15^th^ to the 30^th^ day after renal ablation, and CKD+ASC+LOS (N=14), that received both the ASC subcapsular injection and the oral treatment with Losartan. Sham-operated animals (N=11) were used as control.

**Supplementary Figure 3:** *In vitro* Experiments. (**A**) Illustrative microphotographs of immunofluorescence of cultured ASC, previously treated with: Normal rat serum (NRS), uremic rat serum (URS) or URS+10μM of Losartan (URS+LOS). Cells positive for PCNA are stained in red. The total number of ASC/mm^2^ in each experimental treatment is represented in the bar graph in **B**, while the number of PCNA+ASC/mm^2^ is represented in **C**.

**Supplementary Figure 4:** Bar graphs of (**A**) IL-1β, (**B**) IL-2, (**C**) IL-6, and (**D**) IL-10 gene expression of *in vitro* ASC stimulated or not with uremic rat serum and treated or not with 10μM of Losartan. a: p<0.05 vs. NRS, b: p<0.05 vs. URS.

**Supplementary Table 1:** Genes and primer sequences. The housekeeping beta-actin (*Bact*) gene was used as an endogenous control of the PCR reaction.

**Supplementary Table 2:** Final parameters analyzed at the end of the study period: Survival rate (SR; %), Body weight (BW; g), Urinary volume (UV; mL), Serum creatinine concentration (Scr; mg/dL), Urinary creatinine concentration (Ucr; mg/dL), Creatinine clearance (CrCl; mg/min/RBSA), Blood urea nitrogen (BUN; mg/dL) and Renal hypertrophy. *: p<0.05 vs. Sham 30d; §: p<0.05 vs. Basal CKD, #: p<0.05 vs. CKD; †: p<0.05 vs. CKD+ASC; ‡: p<0.05 vs. CKD+LOS.
